# A Man's Struggle With Idiopathic Intracranial Hypertension: A Unique Case Study

**DOI:** 10.7759/cureus.43735

**Published:** 2023-08-19

**Authors:** Obada A Al Jayyousi, Saleh A Ba-shammakh, Hasn M Haj-Freej, Mohammad E Abu-Hussein, Abdulrahman M Al-bourah

**Affiliations:** 1 General Practice, Jordan University of Science and Technology, AL Ramtha, JOR; 2 Department of General Surgery, The Islamic Hospital, Amman, JOR; 3 Surgery, Jordan University of Science and Technology, AL Ramtha, JOR; 4 Internal Medicine, Jordan University of Science and Technology, AL Ramtha, JOR; 5 Department of Internal Medicine, The Islamic Hospital, Irbid, JOR

**Keywords:** obesity, diabetes mellitus, surgical intervention, lumbar puncture, acetazolamide, visual impairment, male, idiopathic intracranial hypertension (iih)

## Abstract

We report a unique case of a 53-year-old male with idiopathic intracranial hypertension (IIH), predominantly affecting overweight young women. The patient, known to have diabetes mellitus, familial Mediterranean fever, and dyslipidemia, presented with blurred vision and throbbing headaches. Clinical examination, brain MRI/MRV, and a lumbar puncture confirmed the IIH diagnosis. Management with acetazolamide improved the patient's symptoms significantly. This case highlights the potential for IIH occurrence in men and underscores the need for early diagnosis and intervention to prevent potential visual impairment, typically more severe in male patients.

## Introduction

Idiopathic intracranial hypertension (IIH), otherwise referred to as Pseudotumor Cerebri (PC), is a neurological disorder characterized by increased pressure within the brain with a normal composition of the cerebrospinal fluid without any underlying organic primary cerebral condition [1]. The predominant demographic for IIH comprises obese women of reproductive age, with men less frequently impacted [2]. Despite the lower incidence rate among men, they often face a higher risk of severe visual complications [3]. This paper presents the case study of a male patient suffering from IIH, where he manifested severe headaches, visual field anomalies, and progressive deterioration of vision. The combination of therapeutic lumbar puncture and acetazolamide medication led to a substantial recovery in his condition.

## Case presentation

A 53-year-old male patient with a known history of diabetes mellitus (DM), familial Mediterranean fever (FMF), and dyslipidemia came to our outpatient ophthalmology clinic. He had been experiencing blurred vision for the past 10 days, which was bilateral. These visual disturbances were coupled with episodes of progressive, throbbing headaches that seemed to intensify with coughing and straining but were partially mitigated by analgesics.

Regarding medical history, the patient had been under consistent management for his existing conditions. His DM was managed with Vildagliptin, Metformin, Glimepiride, and Insulin Degludec, while his dyslipidemia was kept in check using Atorvastatin. Colchicine was his routine medication for FMF.

Upon arrival, the patient was fully conscious, alert, and oriented. His vital signs were stable, with T: 36.6 C, HR: 75 BPM, BP: 120/80 mmHg, O2 saturation: 98%, and a weight of 98 kg (BMI = 32.2 kg/m2). The physical examination revealed no abnormalities in his sensory and motor functions, with all cranial nerves intact. The patient's deep tendon reflexes were preserved, peripheral pulses were present, and no ulcers were observed on the lower extremities.

**Figure 1:**
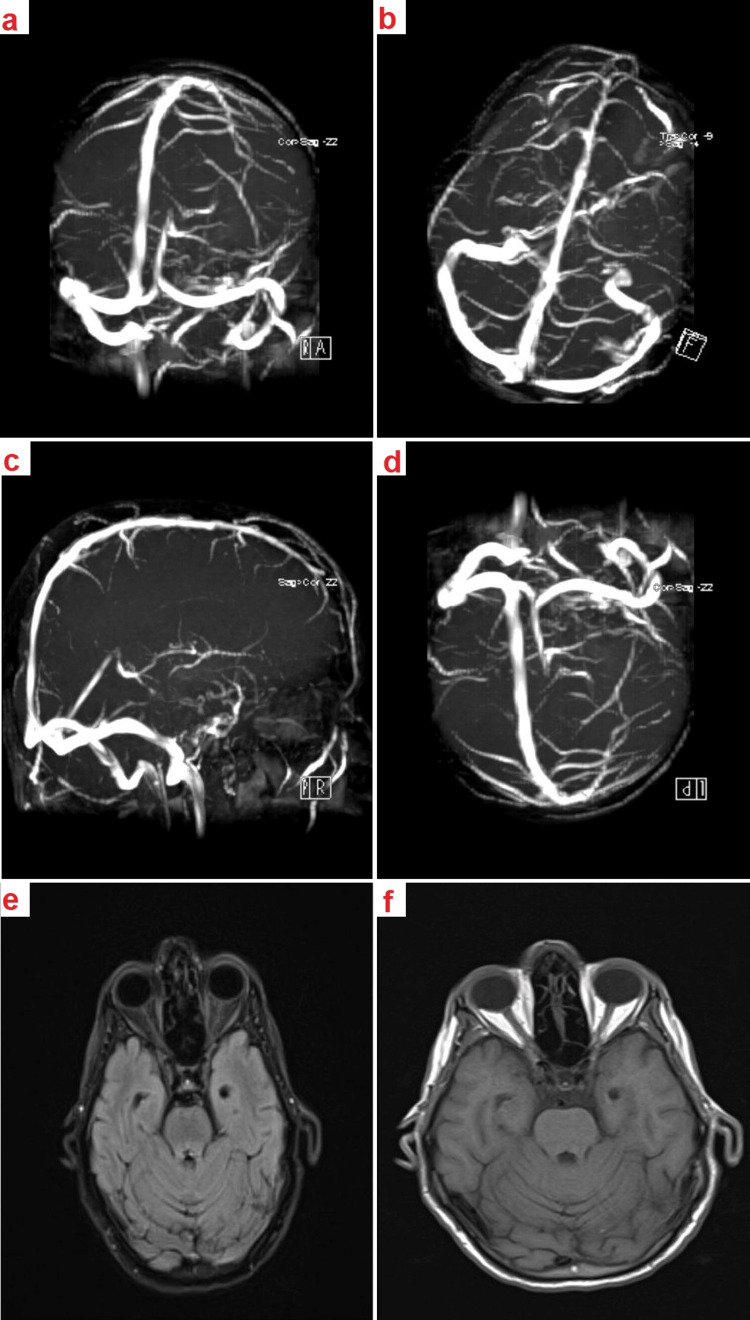
Brain Magnetic Resonance Venography (MRV) and Magnetic Resonance Imaging (MRI) a-d: Brain MRV sequences showcasing patent venous sinuses with no evidence of venous sinus thrombosis or stenosis. e-f: Brain MRI images presenting a homogenous appearance of the brain tissue with no indications of increased intracranial hypertension (ICH) or other abnormalities.

Upon fundoscopy, a thorough examination of the patient's eyes showed no signs of papilledema. Notably, mild retinal revascularizations were observed, attributable to chronic diabetes mellitus. After ruling out other ophthalmic conditions, the patient was promptly referred for a neurological consultation. Follow-up investigations, including brain MRI and MRV, were unremarkable (Figures 1a-1f).

However, a lumbar puncture (LP) was performed, which showed normal CSF parameters (Table 1) *except for an elevated opening pressure (38 mmHg)*. Therapeutic LP was conducted to alleviate the pressure, drawing 18 cc of CSF, which resulted in a decreased pressure not exceeding 20 mmHg and an immediate improvement in the patient's blurred vision.

**Table 1:** Lumbar Puncture (LP) Parameters Shows the lumbar puncture parameters for the patient, with all results falling within the standard reference range. CSF: Cerebrospinal Fluid, mg/dl: Milligrams per deciliter, WBC: White Blood Cells, RBC: Red Blood Cells

Parameters	Value	Reference Range
CSF Protein	57 mg/dl	15-60 mg/dl
CSF glucose	80 mg/dl	50-80 mg/dl
CSF WBC	0	0-5 cells/mm³
CSF RBC	0	0 cells/mm³

In addition, a motor nerve conduction study revealed diffuse, moderate sensorimotor axonopathy consistent with chronic DM. Based on the findings, the patient was diagnosed with idiopathic intracranial hypertension (IIH), having ruled out other potential causes.

For management, the patient was put on Diamox 250 mg twice daily for two weeks, which was later increased thrice daily. The patient returned for follow-up appointments at one, three, and six months, reporting no new complaints. Throughout these visits, his vision and headache remained controlled, indicating an effective response to the treatment. The patient continues to be monitored regularly to ensure early detection of any recurrence or possible complications.

## Discussion

Idiopathic intracranial hypertension (IIH), a condition typically seen in overweight females aged 15-40, presents uncommonly in males [4]. The epidemiological studies indicate a higher prevalence in females, with women eight times more likely to experience IIH [5]. However, incidences in males, though rare, are noteworthy and vary based on regional obesity rates [6].

The clinical manifestations in males parallel those in females, encompassing symptoms of increased intracranial pressure (ICP) [7]. Interestingly, men are inclined to suffer from more severe vision loss, demonstrating higher rates of visual acuity and visual field defects [4,8]. A rapid presentation between the onset of symptoms and diagnosis has also been noted in males, possibly due to more pronounced vision loss at presentation [3]. In line with these findings, men often necessitate surgical intervention for vision impairment [4].

According to the literature, few males have been reported to have IIH [4,8]. The diagnosis involves excluding any intracranial pathology using modern imaging techniques like MRI or CT and performing a lumbar puncture to measure CSF pressure [7]. In patients diagnosed with IIH, treatment aims to preserve vision and manage ICP [1].

Despite the benefits of weight loss in alleviating IIH symptoms, it alone is inadequate to decrease ICP within the required timeframe for those with visual field deficits [1]. First-line treatment usually involves a carbonic anhydrase inhibitor like acetazolamide to manage ICP [1]. However, patients experiencing accelerated vision impairment might necessitate surgical procedures, such as optic nerve sheath fenestration (ONSF) or shunting, particularly if pharmacological approaches prove ineffective [9]; further exploration is crucial to understand the potential advantages of prompt surgical measures.

Our case presents a middle-aged 53-year-old male exhibiting common symptoms of idiopathic intracranial hypertension, such as headache, nausea, vomiting, and brief visual disturbances, who was diagnosed with the disorder despite its unclear underlying mechanisms [2,10,11]. The criteria to diagnose idiopathic intracranial hypertension (IIH) encompass evidence of increased intracranial pressure (ICP), routine neurologic evaluations yielding normal results, elevated ICP alongside unaltered cerebrospinal fluid (CSF) composition, and neuroimaging analyses ruling out other potential reasons for increased intracranial pressure [10,12,13].

Management includes lumbar puncture and pharmacological treatments like carbonic anhydrase inhibitors or, in non-responsive cases, venous stenting or surgery [11,13]. Given the risk of blindness in a small percentage of patients with post-procedure complications, a more precise understanding of IIH pathogenesis is necessary to improve patient outcomes [11,13].

While IIH appears less frequently in men, it can lead to substantial loss of vision, a condition that typically does not respond favorably to non-surgical therapies. As a result, the recommendation is to consider surgical procedures at an earlier stage for male patients experiencing swift deterioration of vision [4,8]. Further research endeavors should aim to assess the effectiveness of early surgical intervention in comparison to medical treatments. As such, even in cases of low incidence in males with low BMI, IIH should be clinically suspected if symptoms suggest the condition. A timely diagnosis can significantly improve the patient's quality of life.

## Conclusions

Although IIH typically manifests in overweight, childbearing-aged women, it can also present in males, often with more significant visual impairment. This case report of a male patient diagnosed with IIH emphasizes the importance of considering this diagnosis even in less commonly affected populations. It underscores the need for prompt diagnostic efforts, such as neuroimaging and lumbar puncture, followed by appropriate treatment strategies to manage symptoms and prevent potential visual loss. Our case demonstrated successful management with acetazolamide. However, the possibility of requiring earlier surgical intervention in males with rapidly deteriorating vision should not be dismissed. Further studies are needed to evaluate the treatment outcomes in this patient demographic.
